# Improved stereo perception in coronary angiography using the X-ray tube as the viewpoint and validation with 3D printed models

**DOI:** 10.1007/s10554-023-02906-x

**Published:** 2023-07-15

**Authors:** Miao Chen, Tianpeng Zhang

**Affiliations:** Department of Emergency, Beijing Friendship Hospital, Capital Medical University, No.95 Yongan Road, Xicheng District, Beijing, 100050 China

**Keywords:** Coronary angiography (CAG), Digital subtraction angiography (DSA), Computed tomography (CT), Coronary heart disease (CHD)

## Abstract

Coronary angiography (CAG) provides two-dimensional images, but a clinician who is experienced in percutaneous coronary interventions can use information from these images to interpret spatial depth and infer the three-dimensional (3D) locations of vessels. We hypothesized that CAG results were equivalent to the mirror image of a coronary artery perspective projection, and a stereo perception could be easily established when the viewpoint of the angiogram was the X-ray tube instead of the detector. To eliminate the influence of heartbeat and respiration, a 3D-printed a coronary artery model was constructed for analysis. The effects of gantry movements during digital subtraction angiography (DSA) on the image were used to identify factors that affected DSA image transformation. Then, based on these factors, DSA imaging was simulated using UG NX software with three methods: (i) a perspective projection with the detector as the viewpoint; (ii) a parallel projection; and (iii) a mirror image of the perspective projection with the X-ray tube as the viewpoint. Finally, the resulting 3D images were compared with the DSA image. Our mirror image of the coronary artery perspective projection that used the X-ray tube as the viewpoint fused precisely with the CAG results and provided exact simulations of all the effects of DSA gantry movements on the DSA image. CAG results were equivalent to the mirror image of coronary artery perspective projection, and the stereo perception was easily established using the X-ray tube as the viewpoint.

**Supplementary Information:**

The online version contains supplementary material available at 10.1007/s10554-023-02906-x.

## Introduction

Clinical studies that demonstrated improvements in percutaneous coronary intervention (PCI) have led to increased clinical use of this technique for patients with coronary heart disease (CHD) [1]. During this procedure, doctors have historically relied on digital subtraction angiography (DSA) to guide the angioplasty and stent placement. However, DSA only provides two-dimensional (2D) images. Thus, PCI operators must rely upon previous experience and haptic feedback to infer vessel location in 3D space, and this may limit the success of this difficult medical procedure [2]. In fact, experienced PCI operators can interpret coronary angiography (CAG) results as 3D by making inferences about spatial depth. However, few studies have examined the establishment of stereo perception during CAG.

A perspective projection is often used to create an image that indicates the 3D shape of an object on a 2D surface. Perspective projection occurs naturally as the central projection of an object onto the human retina, with the projection center as the viewpoint, and this projection is therefore most realistic. Thus, objects in a perspective projection appear smaller as their distance from the viewpoint increases, and this allows the viewer to interpret depth information [3]. Therefore, it is necessary to determine the correct viewpoint (projection center) to correctly comprehend spatial depth information when establishing stereo perception from a perspective projection [4].

Parallel projection can also be used to map 3D points onto a 2D surface. In parallel projection, the lines of sight from the object to the projection plane are parallel. Thus, lines that are parallel in 3D space remain parallel in the 2D projected image. Parallel lengths at all points are of the same scale, regardless of the distance from the model to the viewpoint. Therefore, to provide accurate measurements, almost all computed tomography (CT) workstations use parallel projection rather than perspective projection to display 3D reconstruction data [5]. However, due to the absence of depth information, the stereo perception from parallel projection is inferior to that from perspective projection.

**Fig. 1:**
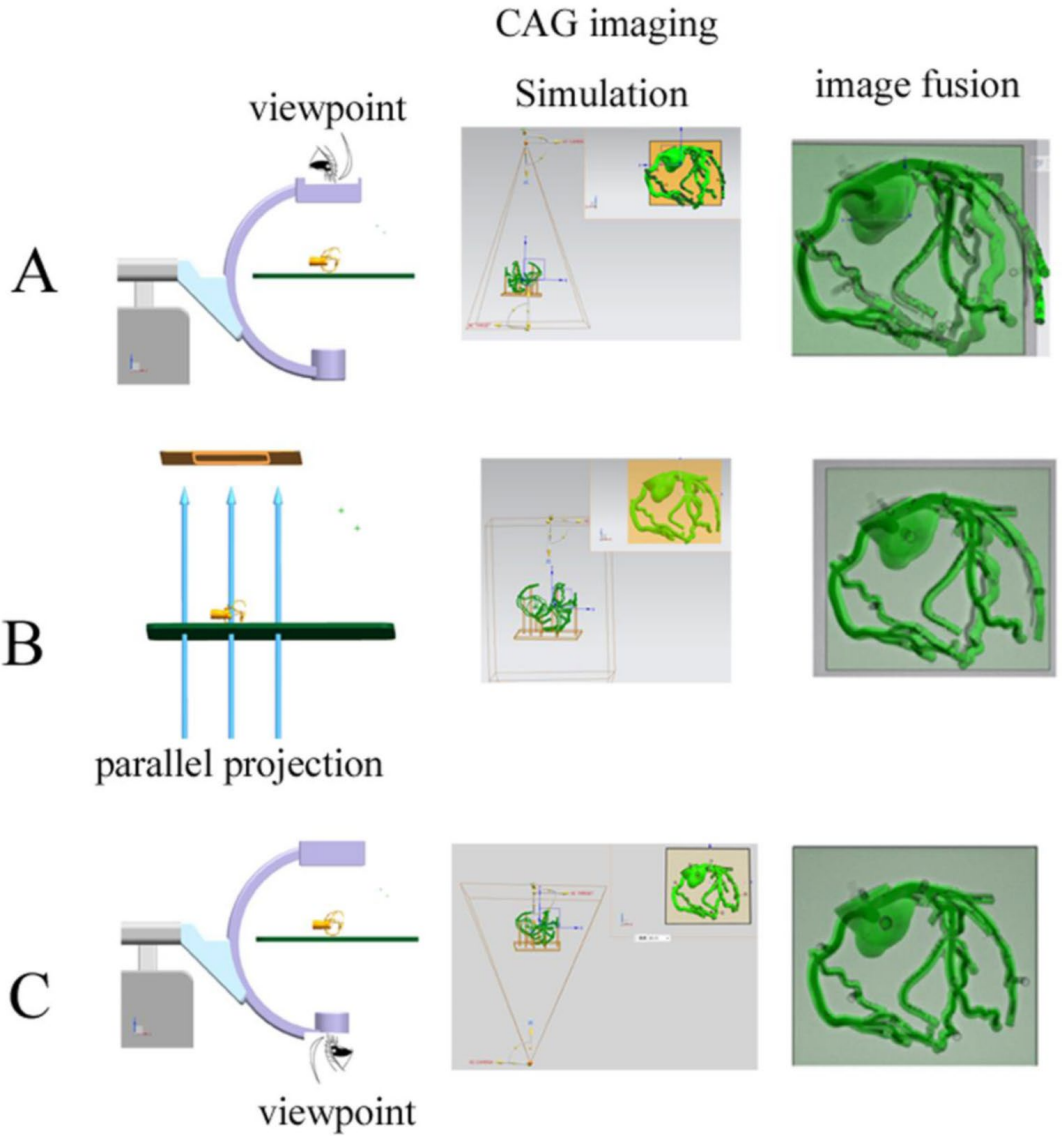
Graphic abstract. **A**, Coronary artery imaging with the detector as the viewpoint. **B**, Coronary artery imaging with parallel projection (no viewpoint). **C**, Coronary artery imaging with the X-ray tube as the viewpoint (**C**). Fusion of images shows the similarity to the CAG results CAG: coronary angiography

In this study, we hypothesized that CAG results were equivalent to the mirror image of a coronary artery perspective projection when using the X-ray tube as the viewpoint of the DSA image instead of the detector. The detector is more commonly used as a viewpoint in clinical practice, and is referred to as the caudal (CAU) or cranial (CRA) view. To test this hypothesis, we used UG NX software (version 11.0) to change the projection mode, projection center, and projection angle of a 3D coronary artery digital model, and then compared the resulting image with the CAG results (Fig. 1). Identifying the correct viewpoint of the CAG results and understanding how CAG results were obtained from the coronary artery can help to establish CAG stereo perception.

## Methods

### Printing the integrated 3D coronary model with a flat base

**Fig. 2:**
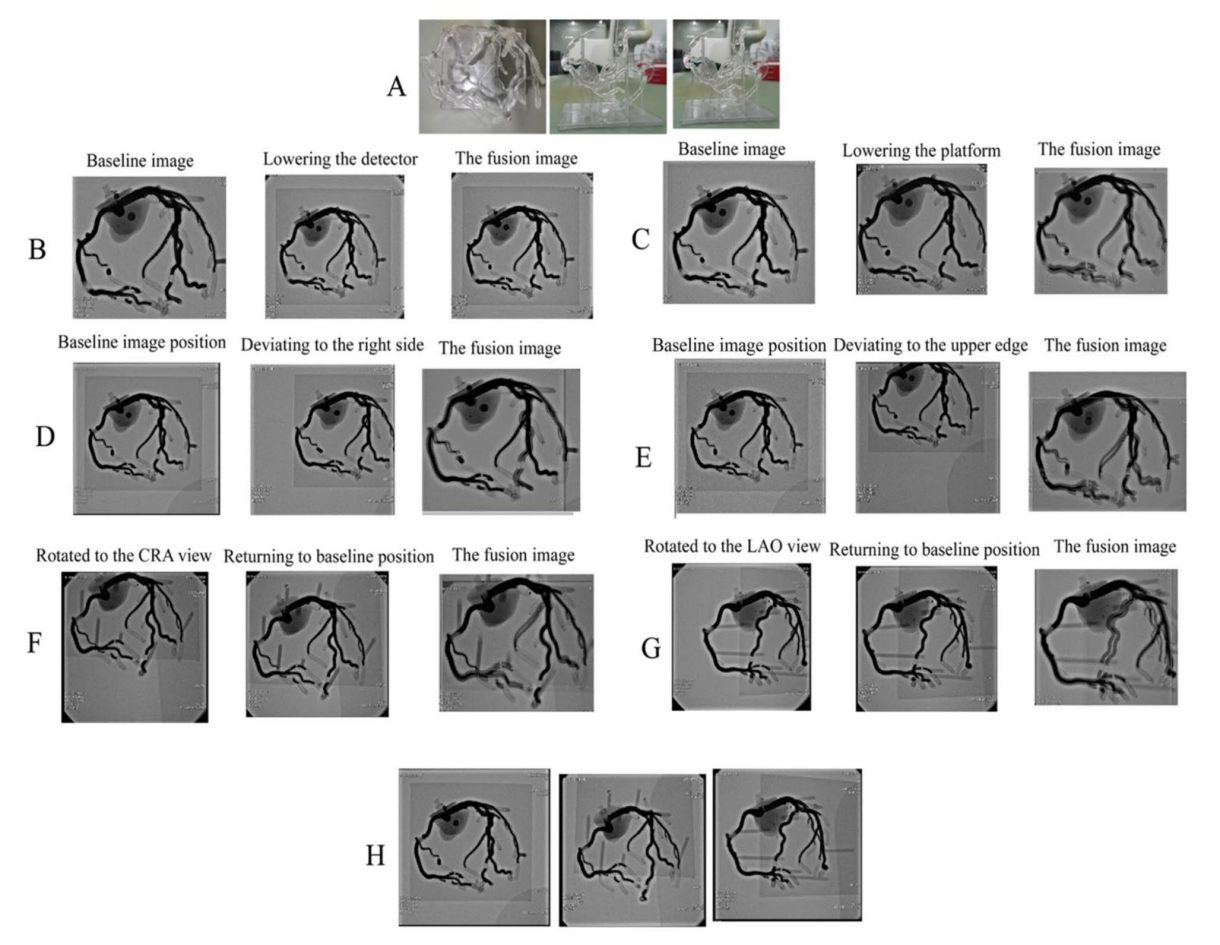
Effects of common DSA gantry movements on the DSA image. Note the image position on each screen. **A**, 3D printed coronary model. **B**, Lowering the detector. **C**, Lowering the platform. **D**, Moving the image to the upper edge of the screen without changing the gantry angle. **E**, Moving the image to the right edge of the screen. **F**, Rotating to the CRA view with the model lower than the isocenter. **G**, Rotating to the LAO view with the model lower than the isocenter. **H**, Rotating to the LAO or CRA view with the model at the isocenter. LAO: left anterior oblique; CAU: caudal; CRA: cranial

To eliminate the influence of heartbeat and respiration and inconsistencies of body position during CT and CAG, a coronary artery model developed from 3D CT reconstruction was obtained from Shanghai Preclinic Medical Technology (China). In this procedure, a digital flat base for the coronary artery digital model was fused using UG NX software (version 11.0) before printing, and then integrally printed using 3D technology (Fig. 2A). Compared with the post-assembly of the coronary model and the flat base, integrated printing can prevent shifts between the coronary model and the flat base (which can easily occur during post-assembly) and thus ensure that the actual position of the printed coronary artery model and the flat base were consistent with the engineered version.

### Effects of DSA gantry movements during PCI on DSA images

The coronary model was placed on the DSA platform. Then, several common DSA gantry movements (changing the height of the platform and detector, shifting the platform horizontally, and changing the gantry angle) were performed during the PCI procedure to identify factors that affect DSA image transformation.

### Simulating DSA imaging using UG NX software

The built-in camera function of the software allows selection of parallel or perspective projection, and can also set the position between the digital model and the projection center, the distance from the projection center to the digital model, and the distance from the projection center to the display screen. Therefore, this software was used to examine DSA image transformation caused by DSA gantry movements by exactly simulating these movements and the corresponding effects on X-ray projections.

### Identifying the rotation axis of the projection center using computer simulations that correspond to the DSA gantry movements

**Fig. 3:**
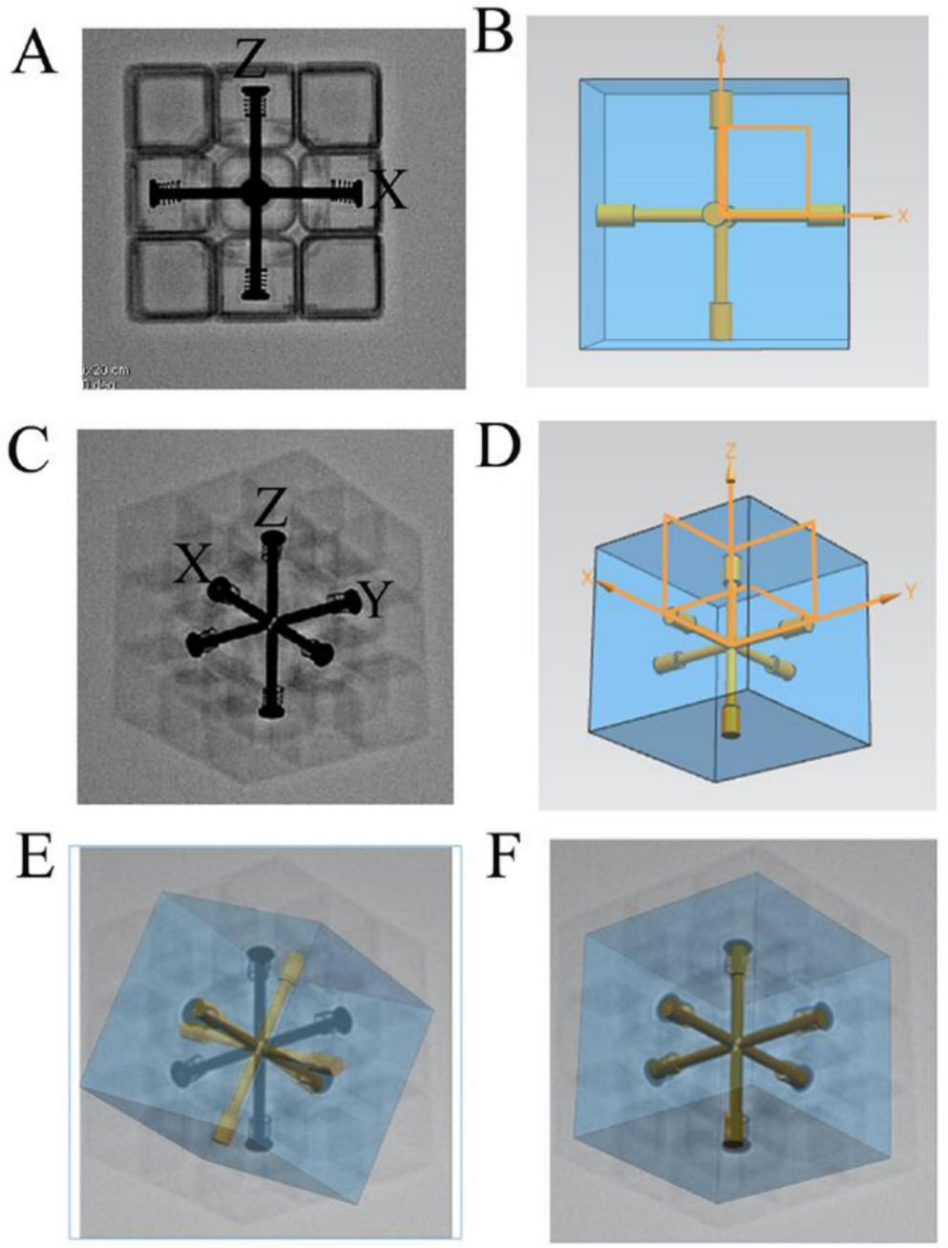
Identifying the rotation axis of the projection center using computer simulation. **A**, DSA image of Rubik’s cube with no gantry angle. **B**, Digital model with no projection angle. **C**, DSA image of Rubik’s cube at RAO28.8 + CRA31.4 view, showing the X, Y, and Z axe. **D**, X, Y, and Z axes in the digital model. **E**, Fusion of the DSA image with the digital model after rotating the projection center around the axis parallel to left-right edges of the display screen, and then around the axis parallel to upper-lower edges. **F**, Fusion of the DSA image with the digital model after rotating the projection center around the axis parallel to upper-lower edges of the display screen, and then around the axis angled with the left-right edges (at an angle equal to the C-arm angle) at the Pivot-arm angle. CRA: cranial; RAO: right anterior oblique

To reduce the effects of other factors and to simply examine the rotation axis, a 3D model was reconstructed using a 3 × 3 Rubik’s cube (Fig. 3B). Placing the cube on the rotational DSA platform (GE Healthcare, United States) allowed clear visualization of the X, Y, and Z axes using X-rays (Fig. 3A). DSA images of the cube with different DSA gantry angles (Fig. 3C) were collected, and a digital model was then established using the UG NX software (Fig. 3D).

### Processing coronary artery digital model projections for image fusion

DSA images of the coronary artery model were collected using different DSA gantry angles. Then, the coronary artery digital model projections were processed using UG NX software, and were overlapped with the DSA images. Three methods were used to process the coronary artery digital model projections for image fusion: (*i*) perspective projection of the digital model using the detector as the projection center; (*ii*) parallel projection, in which all CT reconstruction data were displayed; and (*iii*) mirror image of the perspective projection using the X-ray tube as the projection center.

## Results

### Effects of common DSA gantry movements on the DSA image

We found that raising or lowering the DSA detector caused no changes in the DSA image (Fig. 2B), but lowering or raising the platform led to obvious changes (Fig. 2C **and Suppl. Video 1**). Furthermore, when the DSA image deviated from the previous position of the screen due to horizontal shift of the platform, it rotated automatically without changing the gantry angle (Fig. 2D and E, and Suppl. Video 2). When the coronary artery model was at the DSA isocenter (the intersection of the C-arm rotation axis and the Pivot-arm), the DSA image maintained its position on the screen regardless of gantry angle (Fig. 2H). For a stationary platform, when the model was lower or higher than the isocenter, the DSA image deviated from the previous position of the screen after gantry rotation. Due to the changing position of this image, it was not identical to the image from the previous position after rotation. However, we eliminated this inconsistency by shifting the platform horizontally, so that the DSA image returned to the previous position (Fig. 2F and G, and Suppl. Video 3). These findings indicated that DSA image transformation is completely determined by DSA gantry angle, the distance of the X-ray tube to the platform, and the position of the DSA image on the screen.

### Use of computer simulation to identify the rotation axis of the projection center

To simulate common movements of the DSA, we first determined the rotation axis of the projection center in the computer simulation that corresponded to that of the DSA gantry. First, with no projection angle, we used UG NX to set the position of the digital model (Rubik’s cube) so that the X axis was parallel to the upper and lower edges of the screen and the Z axis was parallel to the left and right edges of the display screen (Fig. 3B). Then, we rotated the projection center two ways according to the recorded gantry angle. In the first method, we performed rotation of the projection center around the axis parallel to left-right edges of the display screen at the Pivot-arm angle, and then rotation around the axis parallel to upper-lower edges at the C-arm angle (Fig. 3E). In the second method, we performed rotation of the projection center around the axis parallel to upper-lower edges of the display screen at the C-arm angle, and then rotation around the axis angled with the left-right edges (an angle that was equal to the C-arm angle) at the Pivot-arm angle (Fig. 3F). Overlapping the projection image with the DSA image showed that the X,Y, and Z axes in the UG NX simulation exactly fused with the X,Y, and Z axes in the DSA image when using the second method.

### Effects of different processing methods on the coronary artery digital model projection

**Fig. 4:**
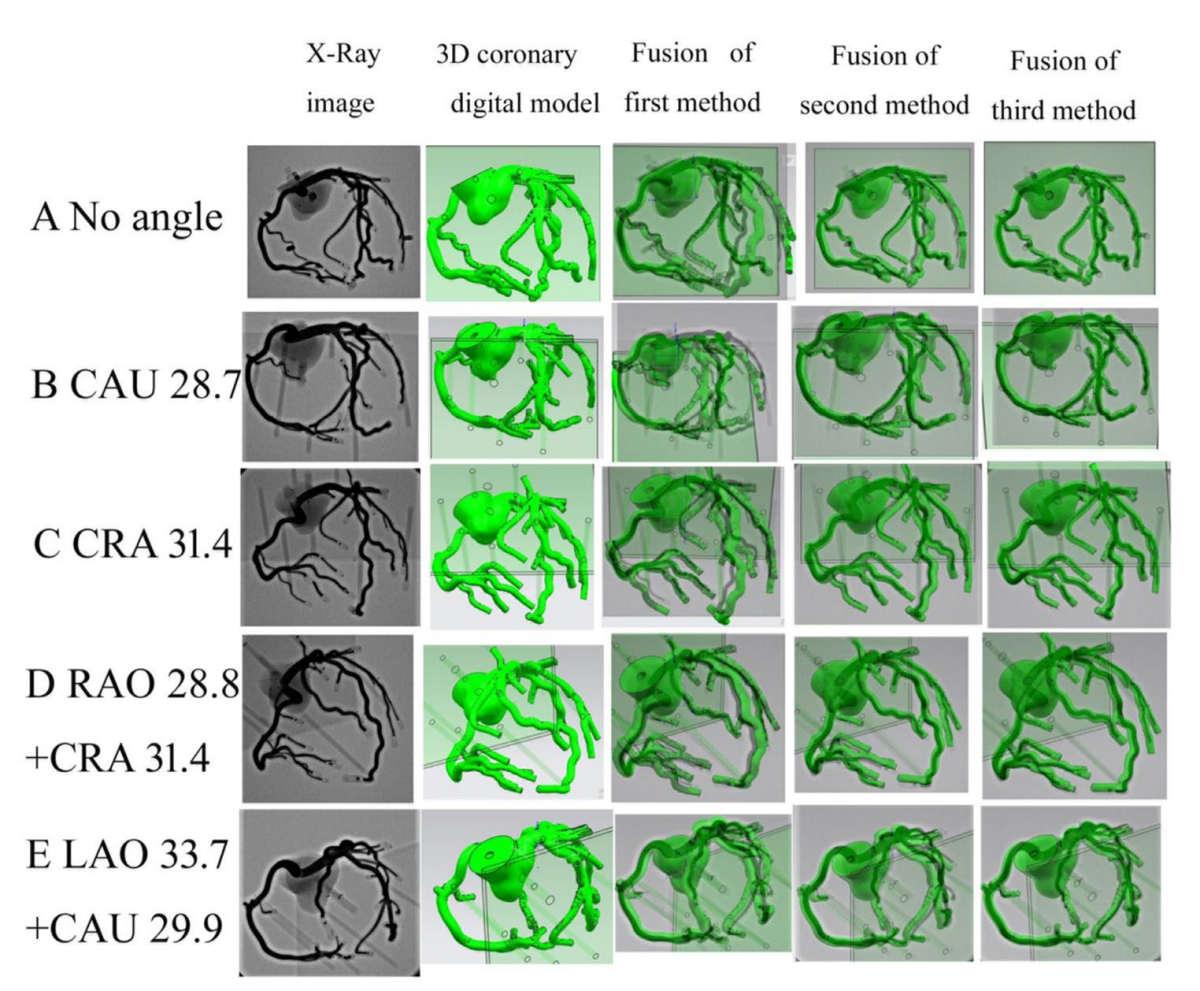
Effects of using three different methods to process the coronary artery digital model projection for image fusion. First method: perspective projection with the detector as viewpoint. Second method: parallel projection. Third method: mirror image of perspective projection with the X-ray tube as the viewpoint. CAU: caudal; CRA: cranial; RAO: right anterior oblique; LAO: left anterior oblique

**Fig. 5:**
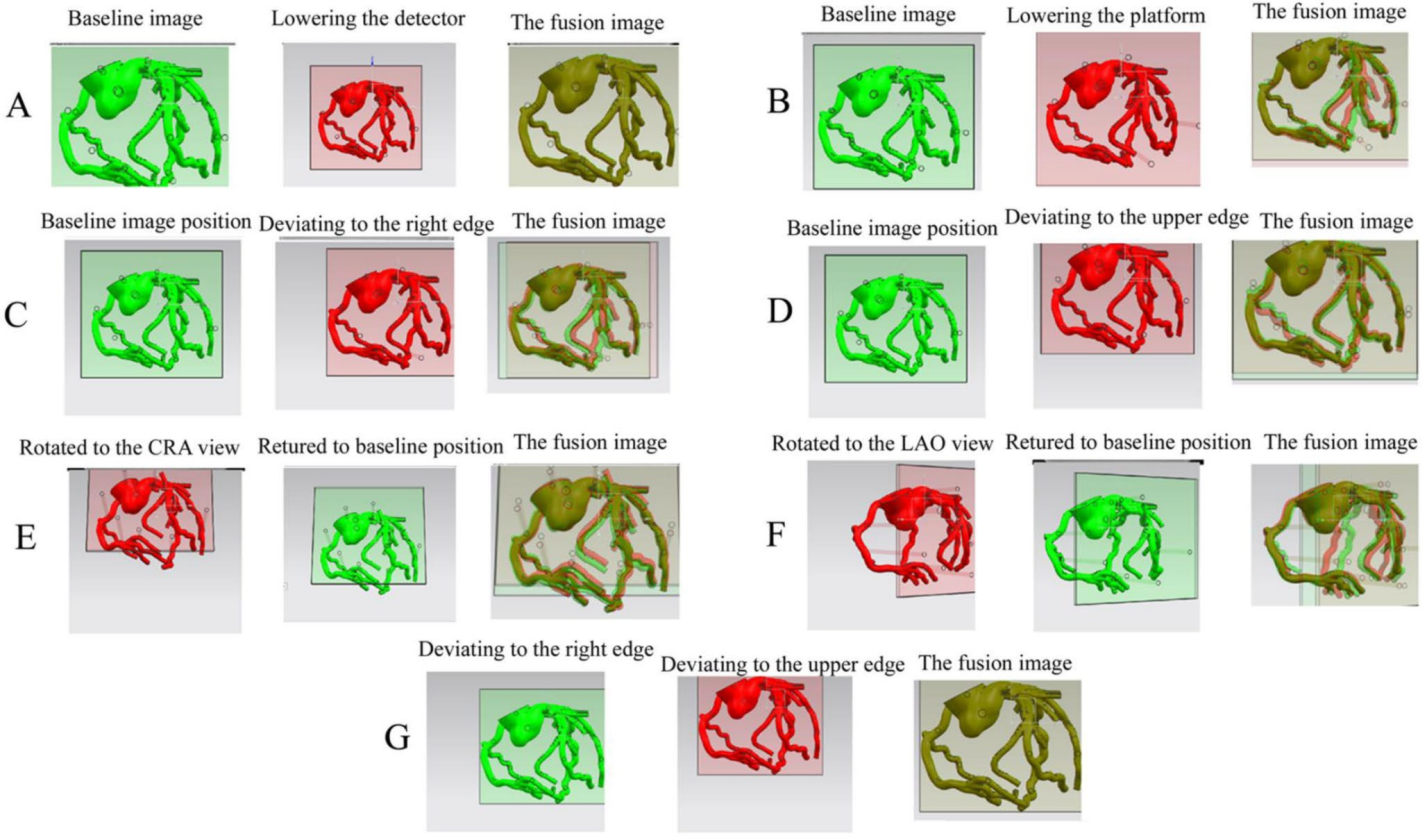
The effects of common DSA gantry movements on the DSA image (simulation using UG NX version 11.0). **A** to **F**, Mirror images of coronary artery perspective projection with the X-ray tube as the viewpoint. **A**, Lowering the detector. **B**, Lowering the platform. **C**, Moving the image to the upper edge of the screen without changing the gantry angle. **D**, Moving the image to the right edge of the screen. **E**, Rotating to the CRA view with the model lower than the isocenter; **F**. Rotating to the LAO view with the model lower than the isocenter. **G**, Moving the image to the right or upper edge of the screen in parallel projection. LAO: left anterior oblique; CAU: caudal; CRA: cranial

Based on these findings, we used UG NX software to set the distance from the center of the perspective projection to the coronary digital model as the distance from the viewpoint (detector or X-ray tube) to the coronary artery model. To assure that the positions of the projected image and DSA image were identical on the screen, we used the software to register the screen center with DSA screen center, and then moved the flat base of the digital model projection so that it precisely overlapped with the flat base of the DSA image. The square shape of the flat base was square made it suitable as a registration reference (Suppl. Video 4). We rotated the projection center at the DSA gantry angle around the rotation axis identified in the simulation above, and then fused the coronary artery digital model projections that were processed using three different methods. The results indicated the third method (mirror image of perspective projection with the X-ray tube as the viewpoint) fused precisely with the DSA image (Fig. 4). Importantly, the effects of common DSA movements on the DSA image were easily observed in the mirror image of the perspective projection created using the UG NX simulation (Fig. 5A **to F**), but this did not occur in the parallel projection (Fig. 5G).

## Discussion

**Fig. 6:**
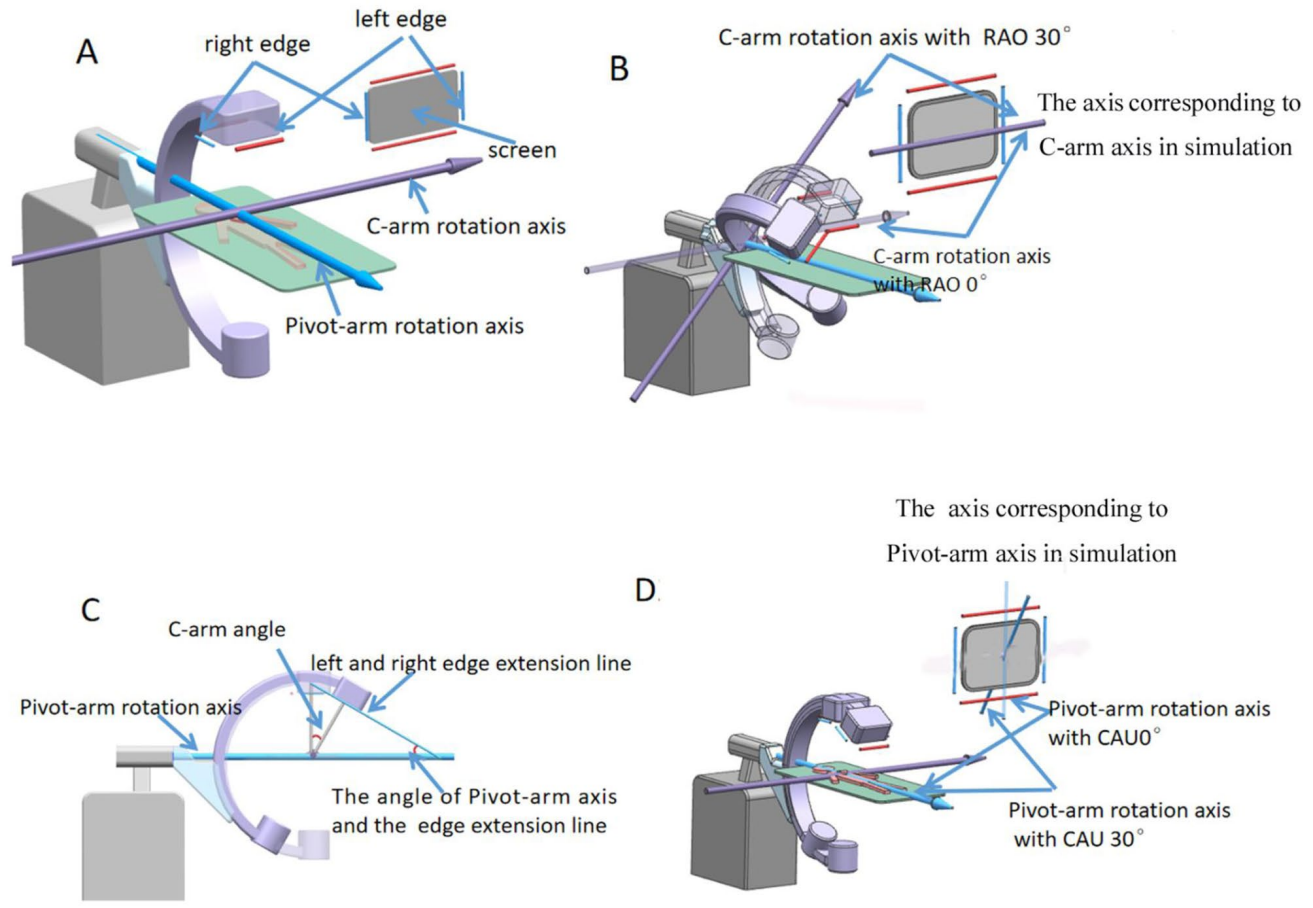
Interpretation of the rotation axis of the projection center in computer simulation corresponding to the DSA gantry. **A**, DSA detector edge is the same as the edge of the screen. **B**, Rotation axis of the projection center in computer simulation that corresponds to the C-arm axis at RAO0 and RAO30. **C**, Pivot-arm rotation axis and the left-right edge of the detector are adjusted according to the C-arm angle. **D**, Rotation axis of the projection center in computer simulation that corresponds to the Pivot-arm axis at CAU0 and CAU30. LAO: left anterior oblique; RAO: right anterior oblique CAU: caudal

Our examination of the effects of common DSA gantry movements indicated that three factors — DSA gantry angle, the distance from the X-ray tube to the platform, and the position of the DSA image on the screen — completely determined image transformation. Our computer simulations found that the rotation axis corresponding to C-arm axis was parallel to the upper-lower edges of the display screen, whereas the rotation axis corresponding to Pivot-arm axis and the left-right edges of the display screen had the same angle as the C-arm. The explanation for this finding may be as follows: The image displayed on the computer screen is the image on the detector, thus the edge of detector is the same as the edge of the screen (Fig. 6A). As the detector is fixed on the C-arm, the C-arm rotation axis is always parallel to the upper-lower edges of the detector, and the rotation axis corresponding to the C-arm axis is also parallel to the upper-lower edges of the screen regardless of the Pivot-arm rotation axis angle (Fig. 6B). Besides, because the C-arm moves relative to the Pivot-arm rotation axis when it rotates, the detector fixed on the C-arm also moves relative to the Pivot-arm rotation axis. As a result, the Pivot-arm rotation axis and the left-right edge of the detector had the same angle as the C-arm rotation (Fig. 6C). Thus, the rotation axis corresponding to Pivot-arm axis and the left-right edge of the display screen also had the same angle as the C-arm rotation (Fig. 6D).

**Fig. 7:**
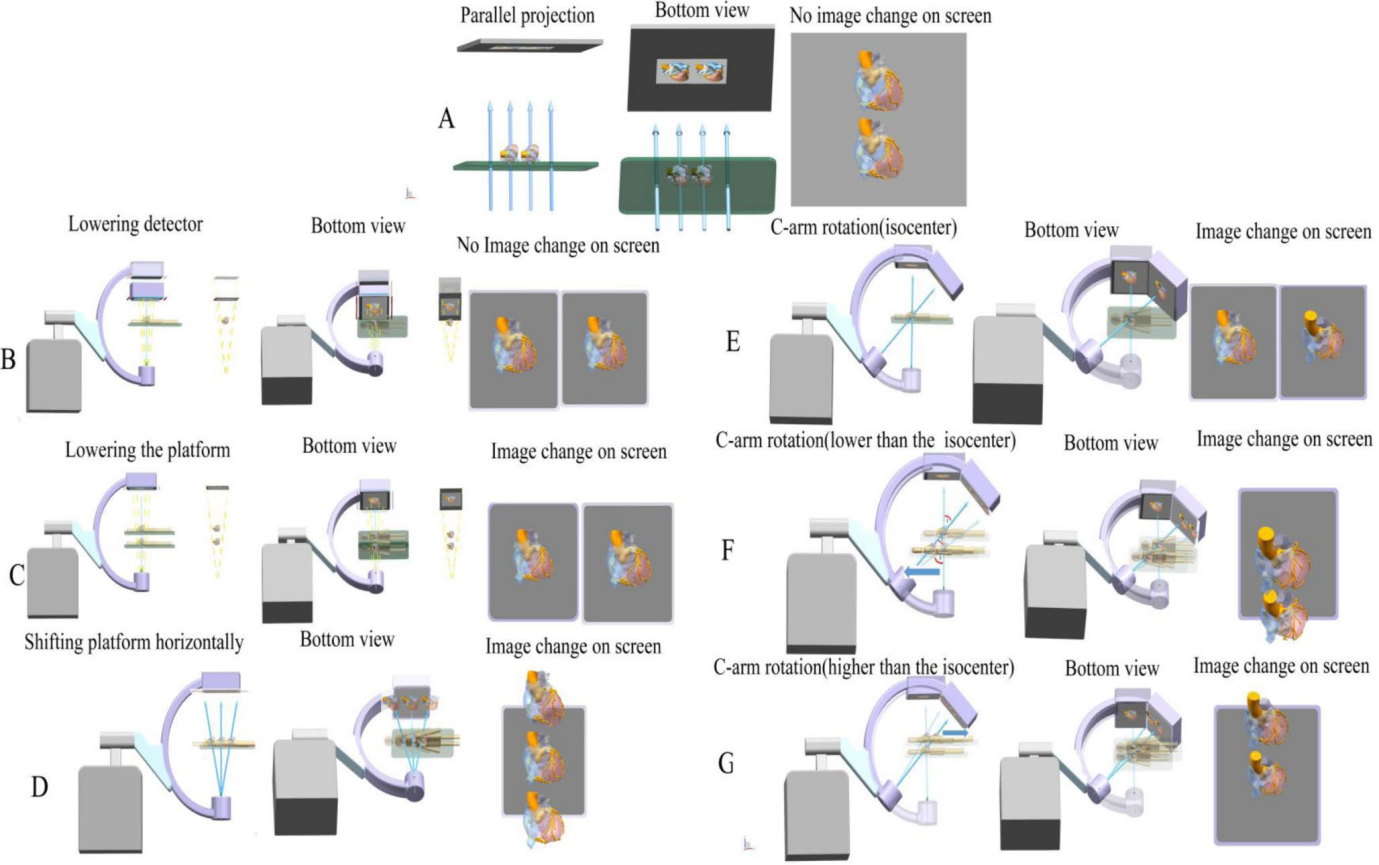
Interpretations of the effects of common DSA gantry movements on the DSA image. **A**, Parallel projection. **B**, Lowering the detector. **C**, Lowering the DSA platform. **D**, Moving the platform horizontally. **E**. Rotating the C-arm with the model at the isocenter. **F**, Rotating C-arm with the model lower than the isocenter. **G**, Rotating the C-arm with the model higher than the isocenter

Our UG NX simulation confirmed that CAG was equivalent to the mirror image of the coronary artery perspective projection with the X-ray tube as the projection center, and for a projection center equal to the viewpoint in a perspective image, the viewpoint of the CAG was X-ray tube. We also unexpectedly found that the relative spatial position of the X-ray tube and the model could be obtained by importing all factors that determined DSA image transformation. Therefore, we interpreted the DSA image transformation using UG NX simulation. Because the model projection image was scaled evenly, no image change occurred when shifting the platform in parallel projection (Fig. 7A) and when lowering the detector in DSA imaging (Fig. 7B). On the contrary, because X-ray lines are derived from the tiny focus of the X-ray tube, when the X-ray tube is closer to the model (due to lowering the DSA platform) the model projection image was more uneven due to increased image distortion [6] (Fig. 7C and Suppl. Video 1). Besides, shifting the platform horizontally changed the DSA image position on the screen, and the X-ray lines from the tube to the model were angled with the previous X-ray lines, and this led to rotation of the projection angle without gantry rotation (Fig. 7D and Suppl. Video 2). When the object is at the DSA isocenter, the X-ray lines derived from the focus of the X-ray tube to the object always point to the fixed position of the detector, regardless of the gantry angle. Thus, the DSA image with the model at the isocenter maintained its position on the screen regardless of gantry angle changes (Fig. 7E). When the model is lower or higher than the isocenter, the X-ray lines derived from the focus of the X-ray tube (viewpoint) to the model point to a position different from the previous position on the screen after gantry rotation (Fig. 7F and G). This caused the DSA projection angle (angle-3 in Fig. 7F) on the screen to be larger than gantry rotation angle (angle 1 in Fig. 7F), consistent with the mechanism described in Fig. 7D. When the X-ray lines pointed to the previous position on the screen, which was achieved by shifting the platform horizontally after gantry rotation, the projection angle (angle 2 in Fig. 7F**)**, which is equal to gantry rotation angle, was then obtained (Suppl. Video 3).

**Fig. 8:**
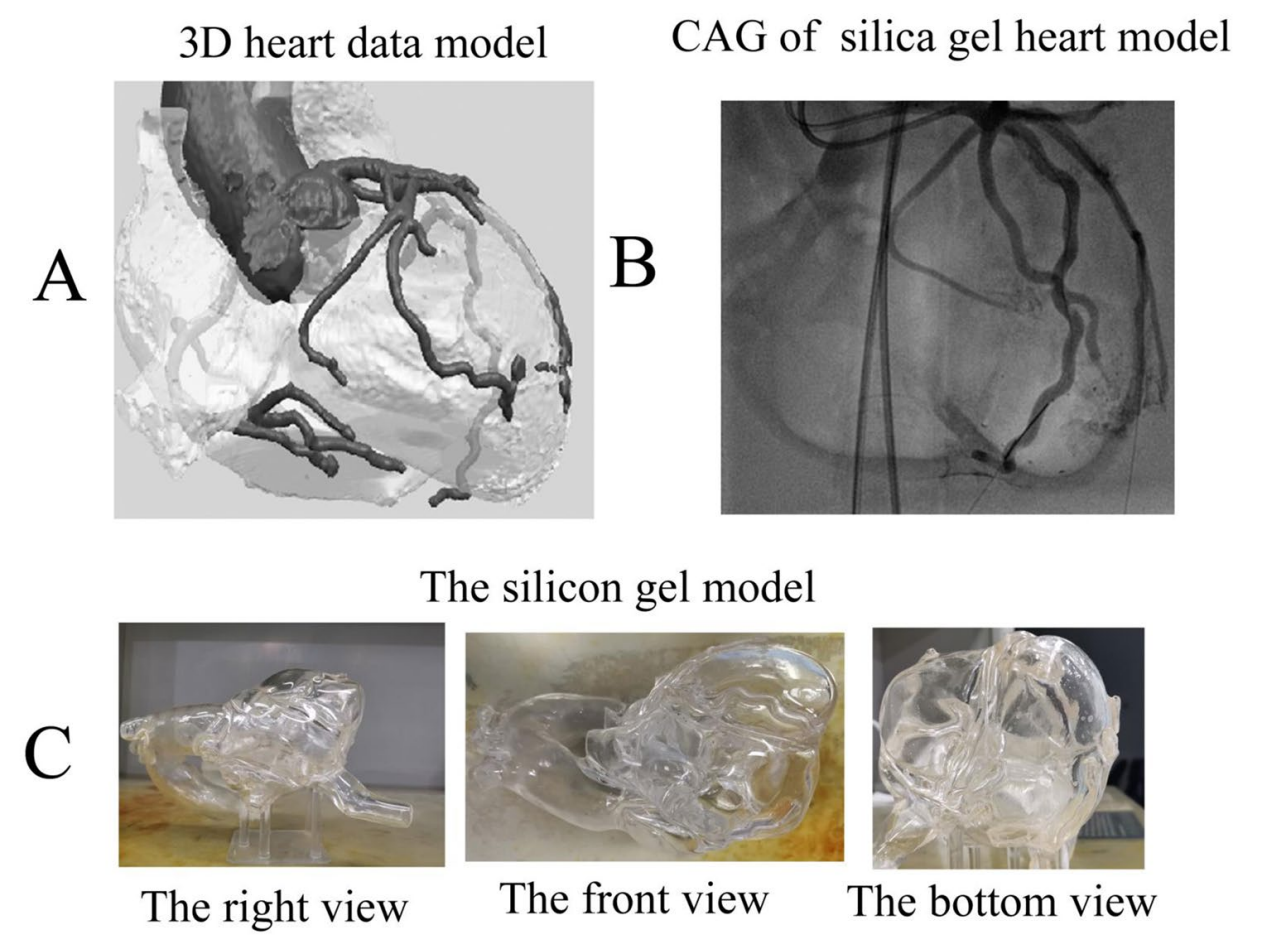
Comparison of the 3D heart model with CAG of the silica gel model. **A**, Heart model developed using UG NX software. **B**, CAG of the silica gel model. **C**, Different views of the silica gel model. CAG: coronary angiography

Because DSA imaging fully conforms to the perspective principle, theoretically it should be easy to form a stereo perception using CAG, but in practice this is not easy. Mistaking the detector as the viewpoint may account for this difficulty. Many doctors consider the CAG to be similar to the coronary artery seen from the detector, and most image fusion software therefore use schemes based on this interpretation [7–10]. Even the commonly used DSA viewpoints, such as the CAU and CRA views, refer to the position of the detector instead of the X-ray tube. We believe that the penumbra effect and the mirror image may explain this mistake. Objects in the real world follow the principle of aerial perspective, in which nearby objects are sharper than distant objects [11]; however, X-ray imaging is the opposite, in that objects near the X-ray tube (viewpoint) are less sharp. This is clearly apparent in our comparison of the digital heart model projection processed by UG NX and the CAG of the silica gel heart model (Fig. 8 and Suppl. Video 5). The reason for this difference is the “penumbra effect” [12]. In particular, because the focus of the X-ray tube is not a simple geometric point, but is an area that varies with the volume of the tube, the resulting X-ray image must contain a penumbra. This penumbra reduces image sharpness. Therefore, an object close to the tube has a large penumbra and is less clear, but an object more distant from the tube has a smaller penumbra and is more clear (Suppl. Figure 1). Because of the different principles underlying of DSA imaging and the aerial perspective, and because the image on the display screen is the mirror image of objects projected on the detector, many doctors inappropriately consider the CAG as the actual coronary artery with the detector as viewpoint [13].

We identified the X-ray tube as the correct viewpoint for CAG and described how CAG can be obtained from a coronary artery model. These findings may help doctors to more easily interpret depth information and establish stereo perception when performing CAG. In addition, because a coronary artery perspective projection that was identical to CAG was achieved using UG NX, this software has potential use as an accurate tool for image fusion that guides PCI operators. This is especially valuable when examining patients with chronic total occlusion (CTO), because a region with total occlusion cannot be visualized using CAG (Suppl. Figure 2).

## Conclusion

We used UG NX simulations to demonstrate that the CAG was equivalent to the mirror image of the coronary artery perspective projection from which stereo perception could be easily established when using the X-ray tube as the viewpoint. These findings may help to improve the PCI skills of doctors and thereby improve the prognoses of CHD patients undergoing PCIs.

### Limitations

A limitation of this study is that all the authors are clinical operators, and have limited experience in medical imaging and computer vision. Nonetheless, despite the extensive research by other professionals in image reconstruction from CAG, there have still been no actual clinical applications of these methods [14]. Another limitation is that removal of interference from factors such as breathing and heartbeat takes too much time and effort. Our current image fusion method is therefore only applicable to a static model, and only applicable to very few cases. Therefore, the image fusion method we described here needs further improvements and evaluations.

### Electronic supplementary material

Below is the link to the electronic supplementary material.

Additional file Fig. 1: Effects of the penumbra on the DSA image. A, Projection center as the viewpoint. B, The X-ray tube is close to the object. C, The X-ray tube is distant from the object.

Additional file Fig. 2: CAG in a normal heart (top) and a heart with chronic total occlusion (bottom). Each patient was asked to hold back after exhalation for the coronary CTA examination and coronary angiography to ensure that the image acquisition was in the end-expiratory phase and eliminate respiratory interference. An R-R interval was extracted from the ECG curve of the CAG image, and each frame (corresponding to a different part of the interval) was then distinguished. The part of the R-R interval for the CTA image was recorded. To eliminate heartbeat interference, the frame corresponding to this recorded part of the R-R interval was chosen for image fusion. For position calibration, we pressed the xiphoid process of the patient to secure one end of the chest cavity, and asked the patient to move the shoulder on the other end of the chest cavity until the DSA image of the thoracic vertebrae accurately fused with the 3D thoracic vertebrae digital model perspective projection.

## Data Availability

The data that support the findings of this study are available from the corresponding author upon reasonable request.
