# Supplementary figures and images for: Improved stereo perception in coronary angiography using the X-ray tube as the viewpoint and validation with 3D printed models
Source: Int J Cardiovasc Imaging. 2023 Jul 15;39(10):2041–50. doi: 10.1007/s10554-023-02906-x (PMC10589187; doi:10.1007/s10554-023-02906-x)

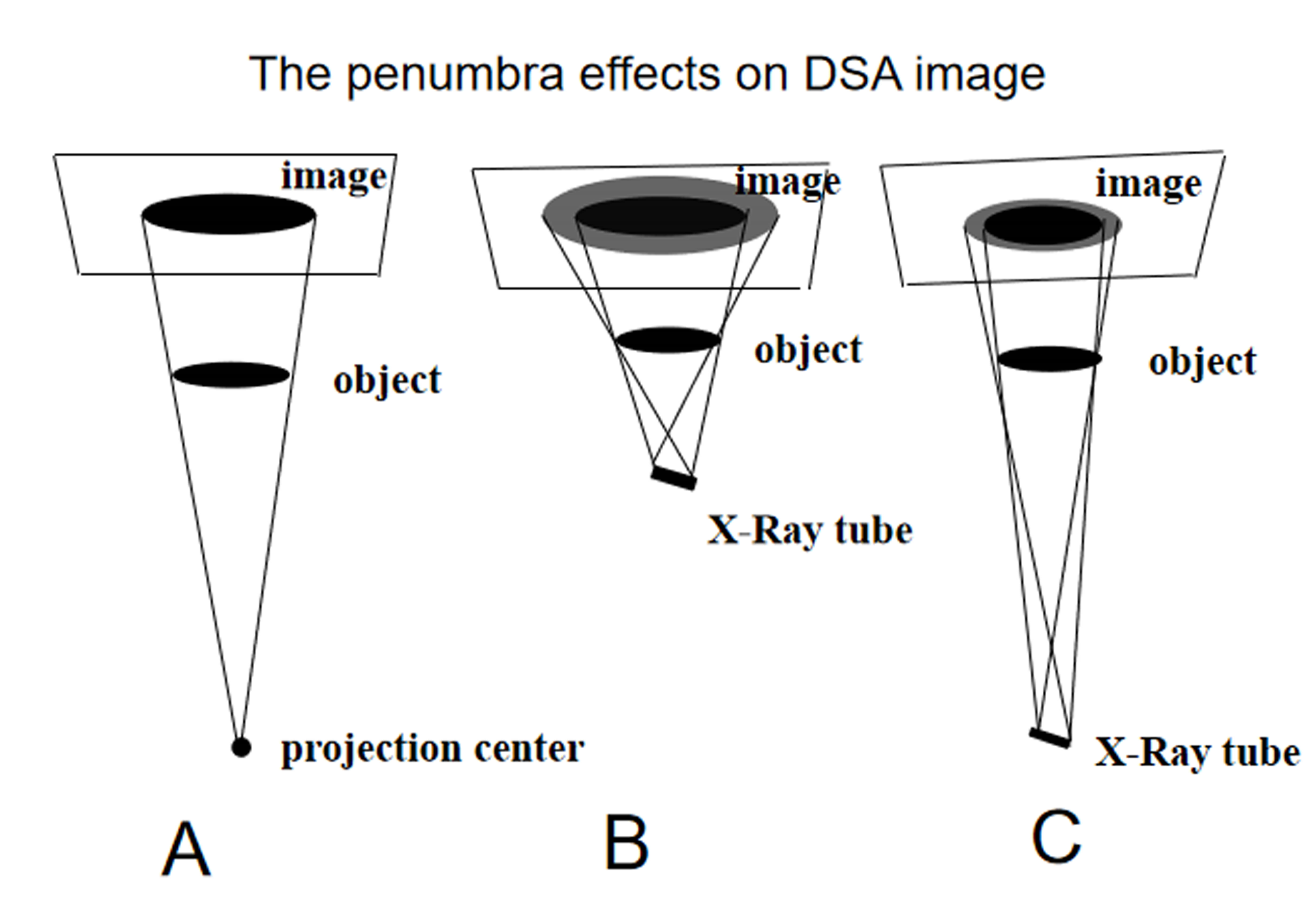

Supplement: Supplementary file 1 — Additional file Fig. 1: Effects of the penumbra on the DSA image. A, Projection center as the viewpoint. B, The X-ray tube is close to the object. C, The X-ray tube is distant from the object. [file 10554_2023_2906_MOESM1_ESM.png]

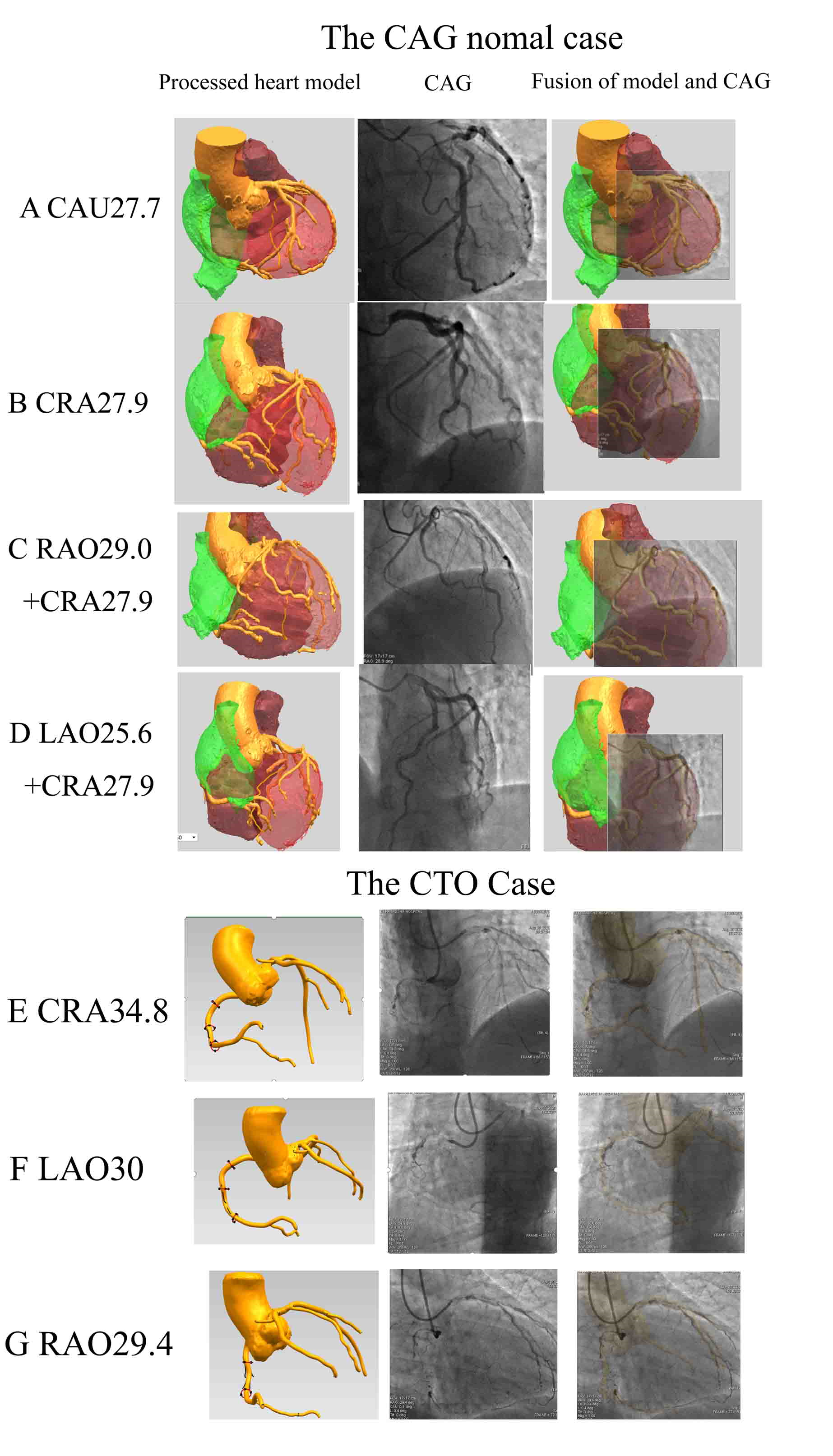

Supplement: Supplementary file 2 — Additional file Fig. 2: CAG in a normal heart (top) and a heart with chronic total occlusion (bottom). Each patient was asked to hold back after exhalation for the coronary CTA examination and coronary angiography to ensure that the image acquisition was in the end-expiratory phase and eliminate respiratory interference. An R-R interval was extracted from the ECG curve of the CAG image, and each frame (corresponding to a different part of the interval) was then distinguished. The part of the R-R interval for the CTA image was recorded. To eliminate heartbeat interference, the frame corresponding to this recorded part of the R-R interval was chosen for image fusion. For position calibration, we pressed the xiphoid process of the patient to secure one end of the chest cavity, and asked the patient to move the shoulder on the other end of the chest cavity until the DSA image of the thoracic vertebrae accurately fused with the 3D thoracic vertebrae digital model perspective projection. [file 10554_2023_2906_MOESM2_ESM.png]
